# Effectiveness of Unguided Internet-Based Cognitive Behavioral Therapy for Subthreshold Social Anxiety Disorder in Adolescents and Young Adults: Multicenter Randomized Controlled Trial

**DOI:** 10.2196/55786

**Published:** 2024-07-22

**Authors:** Kazuki Matsumoto, Sayo Hamatani, Kiko Shiga, Kiyoko Iiboshi, Makiko Kasai, Yasuhiro Kimura, Satoshi Yokota, Katsunori Watanabe, Yoko Kubo, Masayuki Nakamura

**Affiliations:** 1 Division of Clinical Psychology, Kagoshima University Hospital Research and Education Assembly Medical and Dental Sciences Area Kagoshima University Kagoshima Japan; 2 Research Center for Child Mental Development University of Fukui Fukui Japan; 3 Department of Child and Adolescent Psychological Medicine University of Fukui Hospital Fukui Japan; 4 Department of Clinical Psychology Faculty of Human Relations Shigakukan University Kagoshima Japan; 5 Clinical Psychology Course Naruto University of Education Tokushima Japan; 6 Department of Welfare Psychology Faculty of Welfare Fukushima College Fukushima Japan; 7 Kochi Kokusai High School Kochi Japan; 8 Department of Psychology Jin-ai University Fukui Japan; 9 Graduate School of Clinical Psychology Kagoshima University Kagoshima Japan; 10 Department of Psychiatry Graduate School of Medical and Dental Sciences Kagoshima University Kagoshima Japan

**Keywords:** adolescent, social anxiety disorder, general anxiety, adolescents, teens, social anxiety, teenagers, internet-based cognitive behavioral therapy, self-help, randomized controlled trials, young adults, cognitive behavioral therapy, anxiety, mental health, students, colleges, universities, social socialize, anxious, fear, mobile phone

## Abstract

**Background:**

Social anxiety disorder (SAD) is a common mental disorder in adolescents and young adults. Early intervention and support could help prevent the development of full-blown SAD. Considering that adolescents with social anxiety symptoms do not prefer face-to-face sessions due to their fear of communicating with therapists, internet-based cognitive behavioral therapy (ICBT) was implemented.

**Objective:**

This study aimed to examine the effectiveness of complete self-help ICBT for subthreshold SAD in high school and college students with no history of mental disorders.

**Methods:**

A multicenter randomized controlled trial designed to demonstrate the objective was conducted from December 2022 to October 2023. Participants were students enrolled at 6 universities and 1 high school. The intervention was a complete self-help ICBT and consisted of 10 text-based sessions that taught cognitive behavioral therapy techniques for social anxiety in youths and young adults. The comparison was a no-treatment condition (control group), which was randomly assigned in a 1:1 ratio by a computer program. A total of 2 psychological scales were used to assess the severity of social anxiety, and 1 psychological scale each was used to measure symptoms of depression, general anxiety, and quality of life.

**Results:**

In total, 77 students were enrolled as study participants. Through the randomization procedure, 38 participants were included in the intervention group, and 39 patients were included in the control group. Results from the analysis of covariance with depression as covariates showed that the participants in the intervention group had significantly reduced symptoms of social anxiety, depression, and general anxiety compared to the control group. The response rate was 61% (19/31) in the intervention group and 24% (9/38) in the control group: odds ratio (OR) 4.97 (95% CI 1.61-16.53; *P*=.003) in the Fisher exact test. The recovery rate was 68% (21/31) in the intervention group and 34% (13/38) in the control group: OR 3.95 (95% CI 1.32-12.56; *P*=.008). The OR for the remission ratio was 2.01 (95% CI 0.64-6.60; *P*=.20) and for the risk of worsening was 0.23 (95% CI 0.002-1.33; *P*=.10), but no significant difference was observed.

**Conclusions:**

The results of this randomized controlled trial show that fully unguided ICBT improves subthreshold SAD in adolescents and young adults. Interpretation of the effectiveness in preventing SAD that meets the diagnostic criteria is limited by sample size and the follow-up period. Future studies should include more extended observations and larger sample sizes in high-risk populations.

**Trial Registration:**

UMIN-CTR UMIN000050064; https://center6.umin.ac.jp/cgi-open-bin/ctr/ctr_view.cgi?recptno=R000057035

## Introduction

### Background

Social anxiety disorder (SAD) is a mental disorder characterized by excessive anxiety and embarrassment in social situations and fear of being negatively evaluated [1]. The lifetime prevalence of SAD is 4% [2], with a prevalence of 2.1% in adolescents and young adults [3]. Anxiety disorders, including SAD, are the most common psychiatric disorders [4], and mild (subthreshold) forms are common and cause significant disability [5]. The onset of SAD often occurs in the early to midadolescent years [6], with most of the patients having the disorder before their 20s [7]. The adolescent years represent a life stage where interpersonal relationships become more complex, leading to a heightened susceptibility to anxiety in social situations. Quite a few young individuals, even those who do not meet the diagnostic criteria, are aware of experiencing social anxiety at levels below the threshold for diagnosis. For example, a previous study reported 280 high school students in Japan found that approximately 20% experienced moderate social anxiety over a period of 3 years [8]. Another research focused on social fears and SAD among Portuguese youth, and 26% reported with intense self-reported social fear [9]. The presence of subthreshold anxiety disorders increases the risk of full-blown anxiety disorders by more than 3-fold [10]. Treating subthreshold anxiety disorders in a timely manner may prevent the onset of full-blown anxiety disorders [11]. When adolescents develop SAD, it can negatively affect their interpersonal relationships, academic achievement, and career development [12]. Intervention for subthreshold SAD is crucial, as it holds the potential to prevent these issues from arising in the first place.

### Cognitive Behavioral Therapy for SAD

Cognitive behavioral therapy (CBT) is one of the most effective treatment approaches for SAD [13]. By modifying the cognitive behavioral cycle that sustains social anxiety [14], a strong therapeutic effect can be achieved [15]. The effectiveness of face-to-face individual CBT has been established not only for adults but also for children and adolescents [16]. However, low rates of in-person CBT and poor access make it challenging to provide long-term treatment [17]. The low prevalence of CBT in Japan (6.2%) can be attributed to a shortage of cognitive behavioral therapists and its high costs in terms of time and finances [18]. Barriers to mental health treatment have been identified via interviews of patients with SAD. These barriers include a lack of awareness of available treatment facilities, fear of self-disclosure, and financial constraints due to a lack of insurance coverage [19]. Additionally, approximately 80% of adults with SAD developed the condition during adolescence [20]. Adolescence is a period of establishing autonomy, and as the scope of social responsibilities expands, young people tend to hesitate to seek support from others [21]. Access to appropriate treatment remains limited, as many youth do not seek help [22]; this problem is further exacerbated by the shortage of mental health services available worldwide [23]. In resource-limited situations, therapeutic approaches must be refined to improve access to treatment. An early intervention to address the mental health needs of youth could lead to rapid recovery or prevent relapse of mental health problems [24].

### Internet-Based CBT for SAD

Internet-based CBT (ICBT) is a promising solution that can overcome physical barriers and time constraints. Many ICBT programs have been implemented, and recently, countries with high smartphone penetration and internet use have begun to validate the effectiveness of self-help ICBT [25,26]. Self-help programs usually have high dropout rates, but the feature of not having to interact with the therapist has been reported to help reduce the dropout rate of patients with SAD and lead to satisfactory treatment completion rates [27,28]. A previous study including individuals with nonclinical anxiety disorders has also confirmed a significant reduction in anxiety symptoms through complete self-help ICBT: 50 cases underwent a web-based ICBT for SAD [29]. Furthermore, conducting ICBT through mobile devices such as smartphones or tablet PCs, as opposed to desktop computers, may enhance therapeutic effects. Social situations eliciting social anxiety are often present outside the home or private spaces, and intervention through mobile devices facilitates the immediate recall and implementation of CBT techniques when confronted with these situations. Unguided ICBT for individuals with SAD using mobile devices has demonstrated a high effect size with a range of Cohen *d*=0.81 when compared to a waitlist group using the Leibowitz Social Anxiety Scale (LSAS) total score [28,30].

### ICBT for Subthreshold SAD

However, there is currently no study examining the effectiveness of unguided ICBT for subthreshold SAD treatment providers. Due to not meeting the diagnostic criteria, individuals with subthreshold SAD may have lower motivation and potentially higher dropout rates in self-help treatment. Moreover, adolescents with mild to moderate symptoms of social anxiety may show a small or limited treatment response. On the contrary, subthreshold SAD may exhibit a higher average treatment response rate, potentially surpassing the 53.6% observed in diagnosed SAD [31], as it lacks the functional impairments such as depression and avoidance frequently observed in patients with SAD [32]. Due to these remaining areas of uncertainty, it is inappropriate to simply extrapolate evidence from diagnosed SAD when considering the applicability of unguided ICBT to subthreshold SAD.

### Objective

The aim of this study was to assess the effectiveness of intervening in widespread subthreshold SAD during adolescence and evaluate the acceptance of unguided, completely self-help ICBT, taking into consideration dropout.

## Methods

### Ethical Considerations

The research protocol underwent review and was approved by the ethics review board of the Clinical Research Management Center at Kagoshima University Hospital (ID 220196). Participants received both oral and written explanations of the study and were enrolled upon expressing their voluntary written consent. For high school participants, written consent was obtained from both the participants and their guardians. The data collected in this study were anonymized and required a code to identify specific individuals.

### Study Design

This study reports based on the CONSORT (Consolidated Standards of Reporting Trials) Statement for Randomized Trials of Nonpharmacologic Treatments [33]. The CONSORT eHEALTH checklist is presented in Multimedia Appendix 1. A multicenter randomized controlled trial (RCT) was conducted from November 2022 to October 2023 in Japan. A research team (KM, MN, and SH) at Kagoshima University Hospital and the University of Fukui designed this RCT. A total of 6 universities and 1 high school were registered as implementation sites in this study. This clinical trial is registered in the University Hospital Medical Information Network Center, and an overview of the research protocol is publicly available (UMIN000050064).

### Participants and Recruitment

Participants were recruited from 6 universities and 1 high school in Japan. In total, 89 students gave informed consent, and the eligibility criteria were confirmed. Of them, 77 students met the eligibility criteria, and they were enrolled in this clinical trial. The participants were randomly assigned to an intervention group (ICBT) and a control group (no treatment). Participants received coupons worth JP ¥5000 (equivalent to US $31.30) as compensation for each of the 2 data submissions. Participants assigned to the intervention group received compensation at baseline and at the start of the intervention. Those assigned to the control group received compensation at baseline and at 10 weeks. High school participants did not receive remuneration, in accordance with their school’s educational policy.

### Eligibility and Exclusion Criteria

High school or college students between the ages of 15 to 25 years, with a total LSAS score≥30 or higher [27], and with their own smartphone were eligible. The exclusion criteria were diagnosis of psychiatric disorders such as depression, history of CBT within 2 years, IQ<85, imminent suicidal risk, and the presence of advanced diseases such as cancer.

### Intervention

Participants assigned to the intervention group were instructed to complete a specific ICBT program entirely through self-help, using their own smartphones. Participants could contact the research team if they had any specific questions. It was recommended to complete 1 module per week, with automatic email reminders sent every Monday morning, prompting participants to engage in the program. If a module was not completed by Friday, participants received an automatic email reminder on Saturday at noon. The first author (KM) developed an unguided ICBT program for subthreshold SAD in adolescents and young adults. The ICBT program was built on an e-learning platform (learningBOX; learningBOX Inc). The ICBT program is based on the Clark and Wells [14] model. The ICBT program consists of 10 sessions of training on effective CBT components for social anxiety. Table 1 shows the treatment modules. The participants in the control group received no treatment; they also were asked to refrain from accessing information about CBT.

**Table 1:** The modules and tasks of the treatment course.

	Module	Task
1	Psychoeducation and case-formulation	Developing an idiosyncratic version of the formulation
2	Examine the function of safety behaviors and self-focused attention	Manipulating safety behaviors and self-focused attention with behavioral experiments
3	Video feedback to correct negative self-image	Updating negative distorted self-images by video feedback in participant’s smartphone
4	Attention training in photographic social situations	Shifting the focus of attention to external information during a conversation or communication
5	Behavioral experiments to test negative predictions and assumptions	Observing the reactions of others immediately after behavioral experiments
6	Opinion survey to follow-up behavioral experiments	Updating negative assumptions about failures in social situations
7	Handling anticipatory worry and postevent rumination	Exploring the advantages and disadvantages of worry and rumination
8	Image description	Updating negative self-images and impressions
9	Schema work	Addressing dysfunctional negative beliefs and assumptions
10	Prevent relapse	Summarizing coping strategies for social anxiety and reflecting on the progress of the treatment course

### Outcomes

#### Primary Outcome

The primary outcome was the severity of social anxiety, as per the self-rated LSAS score. The LSAS was developed to measure social anxiety [27]. The LSAS contains 24 social situations related to “fear or anxiety” and “avoidance” that the participants are asked to rank on a 4-point scale, with 0=never and 4=severe. The Japanese version of the LSAS has demonstrated reliability and validity and is widely used in clinical and research settings in Japan [34]. Response to the ICBT was defined as a decrease in the LSAS by 28% or more [35]. Remission was defined as an LSAS total score<35, as specified in a previous ICBT study of SAD conducted in Hong Kong [36]. Conversely, to assess the risk of exacerbating social anxiety, we defined worsening as an increase in LSAS of 28% or more.

#### Secondary Outcomes

The secondary outcomes were the scores on 4 self-administered scales: the Social Phobia Inventory (SPIN) is a self-rating scale that measures 3 characteristic aspects of SAD: fear, avoidance, and physiological arousal [37]. In the Japanese version of the SPIN, 17 questions are answered on a 5-point scale, with 0=not applicable at all and 4=strongly applicable [38]. The Patient Health Questionnaire-9 (PHQ-9) was developed to measure depressive symptoms [39,40]. The first 9 items of the PHQ-9 assess how often various depressive symptoms have occurred in the last 2 weeks. The Generalized Anxiety Disorder-7 (GAD-7) scale was developed to measure anxiety levels [40,41]. The first 7 items of the GAD-7 assess how often various anxiety symptoms have occurred in the last 2 weeks. The EQ-5D-5L measures the quality of life (QOL) values for calculating quality-adjusted life years (QALYs) in the economic evaluation of medical technologies [42,43]. In the EQ-5D-5L, the responder answers questions on 5 dimensions directly related to QOL (mobility, self-care, usual sensitivity, pain or discomfort, and anxiety or depression). Each dimension has 5 levels: no problems, slight problems, moderate problems, severe problems, and extreme problems. The QALY ranges from 0 to 1.0, with 0=dead and 1.0=in perfect health. We also calculated the recovery rates of the Improved Access to Psychotherapy program and improvement (recovery) rates considering total scores in SPIN<19 and PHQ-9<10, simultaneously for comparison with a UK sample [44].

### Sample Size

The sample size was calculated to be 78 using G*Power (version 3.1.9.7; The G*Power Team), which is a free statistical analysis software [45,46]. The required sample size of this RCT was estimated to be 52, considering a 2-tailed significance level of .05, a power of 80%, and an estimated effect size of 0.8 [30]. The required sample size above was calculated considering the 50% dropout rate of previous studies with unguided ICBT [47].

### Randomization

Randomized allocation was conducted by the minimization method with a randomization generator [48]. In randomization, LSAS total score <50 or ≥50, sex (male or female), and facilities were used as adjustment factors.

### Blinding

Blinding was not implemented.

### Statistical Analyses

Missing values were imputed using the *mice* package in R (version 4.3.2; R Foundation for Statistical Computing), a free statistical software package [49]. Demographic data at baseline were described, and between-group characteristics were analyzed using independent sample 2-tailed *t* tests. To investigate significant differences in the change of scores between groups for both the primary and the secondary outcome measures, an analysis of covariance (ANCOVA) was conducted. A covariate factor was the severity of depression determined by measuring the PHQ-9 total score at baseline. For treatment response ratio, remission ratio, and recovery ratio, Fisher exact test was performed, and odds ratios (ORs) with 95% CIs were calculated. Analyzing the risk of worsening social anxiety measured in LSAS involved the calculation of risk ratio, relative risk reduction (RRR), absolute risk reduction (ARR), and number needed to treat (NNT). Statistical analyses were implemented by using R (version 4.3.2). A significance level of .05 was used for all analyses.

## Results

### Recruitment

**Figure 1:**
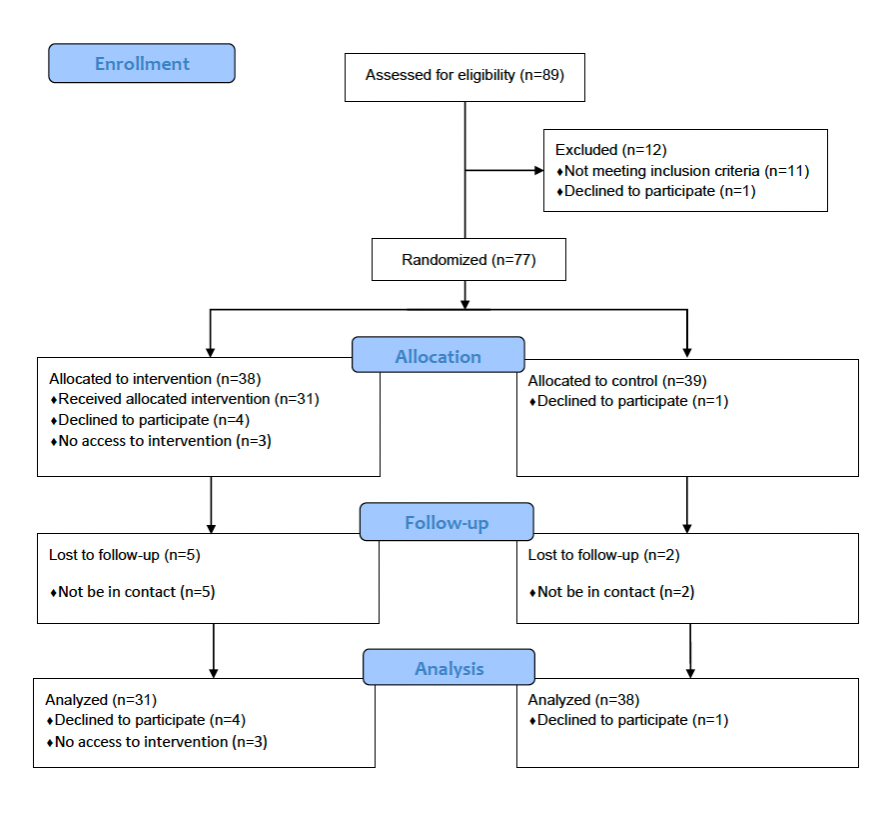
CONSORT (Consolidated Standards of Reporting Trials) flow diagram.

Among the 38 participants assigned to the intervention group, 4 declined research participation after the preintervention assessment. A total of 31 participants completed the ICBT program at least once, while 3 did not access the intervention program at all. Therefore, the dropout rate from this ICBT program was 9% (3/34), the implementation rate of ICBT was 91% (31/34), and the completion rate was 65% (22/34). Of the 39 participants assigned to the control group, 1 declined research participation, and data on outcomes could not be obtained for 2 students due to unavailability. Following the predetermined analysis plan, the statistical analysis included data from 31 participants in the intervention group and 38 in the control group. Figure 1 shows the participant flow diagram.

### Demographical and Characteristic Data

Table 2 shows the demographics, clinical characteristics, and baseline outcomes of the participants included in the analyses. The level of depression, measured by the PHQ-9, in the control group, was significantly higher than the intervention group at baseline; however, clinically, both groups fell within the range of “mild depressive symptoms” (total score 5-9) [50]. No significant differences were observed in other outcomes.

**Table 2:** Demographical and characteristic data, and the total scores of outcomes at baseline.

Demographical and characteristic data	ICBT^a^ group (n=31)	Control group (n=38)	*P* value (unpaired *t* test)
Sex (female), n (%)	20 (65)	25 (66)	N/A^b^
Age, mean (SD)	21.61 (2.06)	21.39 (2.32)	.68
LSAS^c^, mean (SD)	58.38 (19.17)	59.08 (20.77)	.88
SPIN^d^, mean (SD)	25.39 (10.27)	27.42 (12.50)	.47
PHQ-9^e^, mean (SD)	4.87 (4.38)	6.16 (4.29)	.05
GAD-7^f^, mean (SD)	4.13 (4.52)	4.21 (3.30)	.31
EQ-5D-5L, mean (SD)	0.9160 (0.079)	0.889 (0.114)	.28

^a^ICBT: internet-based cognitive behavioral therapy.

^b^N/A: not applicable.

^c^LSAS: Liebowitz Social Anxiety Scale.

^d^SPIN: Social Phobia Inventory.

^e^PHQ-9: Patient Health Questionnaire -9.

^f^GAD-7: Generalized Anxiety Disorder-7.

### Outcomes and Estimation

#### Primary Outcome

**Figure 2:**
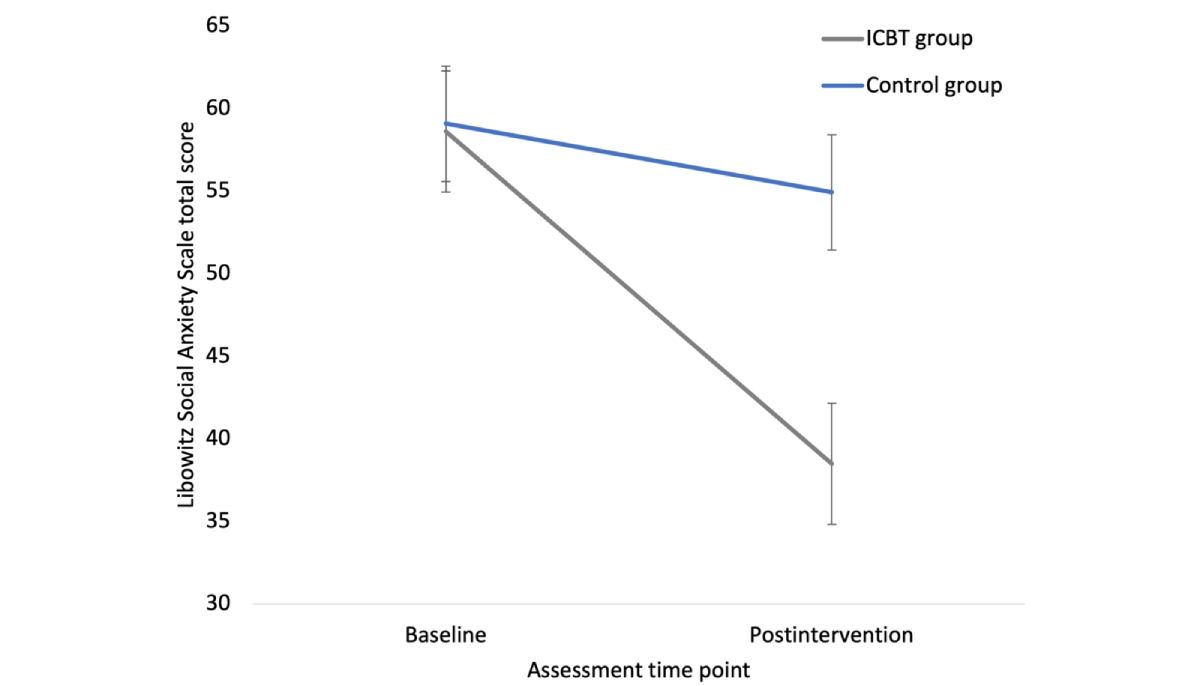
Liebowitz Social Anxiety Scale total score. The graphic presents mean and 95% CI. ICBT: internet-based cognitive behavioral therapy.

The reduction in LSAS total score from baseline to postintervention assessment was significantly greater in the intervention group compared to the control group by 11.62 (95% CI 1.67-21.56; *F*_1,66_=3.91; *P*=.02). Figure 2 presents the change in LSAS total score from baseline to postintervention assessment. The change in LSAS total score from preintervention to postintervention assessment in the intervention group was significantly larger than that in the control group by 9.39 (95% CI 1.31-17.48; *F*_1,66_=4.65; *P*=.01).

#### Secondary Outcome

Table 3 shows the analyses for primary and secondary outcomes in ANCOVA.

**Table 3:** Difference of score in outcomes, *P* value, and effect size.

Outcome and evaluation point		ICBT^a^ group, mean change (SD)		Control, mean change (SD)		*F* test (*df*=2, 66; ANCOVA^b^)		*P* value (ANCOVA)	Effect size (Hedge *g*)
**LSAS^c^**									
	Baseline to postintervention assessment	16.90 (22.63)	4.16 (17.38)		3.91		.02		0.64
	Preintervention to postintervention assessment	14.90 (14.86)	N/A^d^		4.65		.01		0.66
**SPIN^e^**									
	Baseline to postintervention assessment	10.87 (11.40)	2.87 (8.69)		5.39		.007		0.80
	Preintervention to postintervention assessment	11.19 (9.33)	N/A		7.23		.01		0.93
**PHQ-9^f^**									
	Baseline to postintervention assessment	0.68 (3.36)	–0.32 (2.85)		1.19		.31		0.22
	Preintervention to postintervention assessment	1.42 (2.98)	N/A		2.96		.06		0.49
**GAD-7^g^**									
	Baseline to postintervention assessment	0.29 (2.98)	–1.03 (3.17)		1.61		.21		0.09
	Preintervention to postintervention assessment	0.97 (2.83)	N/A		4.15		.02		0.32
**EQ-5D-5L**									
	Baseline to postintervention assessment	–0.0299 (0.09)	0.0079 (0.09)		1.61		.21		0.003
	Preintervention to postintervention assessment	–0.0301 (0.11)	N/A		1.41		.25		0.0004

^a^ICBT: internet-based cognitive behavioral therapy.

^b^ANCOVA: analysis of covariance.

^c^LSAS: Liebowitz Social Anxiety Scale.

^d^N/A: not applicable.

^e^SPIN: Social Phobia Inventory.

^f^PHQ-9: Patient Health Questionnaire-9.

^g^GAD-7: Generalized Anxiety Disorder-7.

The reduction in SPIN total score from baseline to postintervention assessment was significantly greater in the intervention group compared to the control group by 8.05 (95% CI 3.02-13.08; *F*_1,66_=5.39; *P*=.007). The change in SPIN total score from preintervention to postintervention assessment in the intervention group was significantly larger than that in the control group by 8.26 (95% CI 3.75-12.78; *F*_1,66_=7.23; *P*=.01).

The reduction in PHQ-9 total score from baseline to postintervention assessment was greater in the intervention group compared to the control group by 1.15 (95% CI 0.41-2.70; *F*_1,66_=1.19; *P*=.31), not significantly. The change in PHQ-9 total score from preintervention to postintervention assessment in the intervention group was larger than that in the control group by 1.75 (95% CI 0.28-3.23; *F*_1,66_=2.96; *P*=.06), not significantly.

The reduction in GAD-7 total score from baseline to postintervention assessment was greater in the intervention group compared to the control group by 1.24 (95% CI –3.05 to 2.79; *F*_1,66_=1.66; *P*=.21), not significantly. The change in GAD-7 total score from preintervention to postintervention assessment in the intervention group was significantly larger than that in the control group by 1.81 (95% CI 0.31-3.33; *F*_1,66_=4.15; *P*=.02).

The reduction in QALY measured by EQ-5D-5L from baseline to postintervention assessment was greater in the intervention group compared to the control group by –0.0388 (95% CI –0.09 to 0.008; *F*_1,66_=1.41; *P*=.25), not significantly. The change in QALY measured by EQ-5D-5L from preintervention to postintervention assessment in the intervention group was larger than that in the control group by 0.0366 (95% CI –0.0120 to 0.0852; *F*_1,66_=1.31; *P*=.28), not significantly.

### Ancillary Analyses

Table 4 presents Fisher exact test results for treatment response rate, remission rate, recovery rate, and risk ratio. The treatment response rate in the intervention group was significantly higher at 61% (19/31) compared to the control group at 24% (9/38; *P*=.003; OR 4.97, 95% CI 1.61-16.53). The recovery rate in the intervention group was substantially higher at 68% (21/31) compared to the control group at 34% (13/38; *P*=.008; OR 3.95, 95% CI 1.32-12.56). Regarding the risk of deterioration, the intervention group had a higher but nonsignificant rate of 6% (2/31) compared to the control group 24% (9/38; *P*=.10).

**Table 4:** Results of Fisher exact test^a^.

Result	ICBT^b^ group (n=31), n (%)	Control group (n=38), n (%)	OR^c^ (95% CI)	*P* value
Response	19 (61)	9 (24)	4.97 (1.61-16.53)	.003
Remission	12 (39)	9 (24)	2.01 (0.64-6.60)	.20
Recovery	21 (68)	13 (34)	3.95 (1.32-12.56)	.008
Worse	2 (6)	9 (24)	0.23 (0.02-1.23)	.10

^a^Definition of clinically significant change: response: a decrease in the Liebowitz Social Anxiety Scale (LSAS) by 28% or more; remission: an LSAS total score<35; recovery: total scores in Social Phobia Inventory<19 and Patient Health Questionnaire-9<10; and worse: an increase in LSAS of 28% or more.

^b^ICBT: internet-based cognitive behavioral therapy.

^c^OR: odds ratio.

### Adverse Events and Harms

No severe adverse events were reported for this study period. Table 5 presents the relative risk, RRR, ARR, and NNT. The results of the RRR imply that ICBT reduces the occurrence of deterioration events by 73% compared to no treatment in the control group. The results of the ARR imply that ICBT can save 17% more adolescents and young adults from deterioration events of social anxiety compared to no treatment. The results of the NNT suggest that unguided ICBT could be required for 5.8 individuals with subthreshold SAD in adolescents and young adults to prevent deterioration in symptoms for 1 individual.

**Table 5:** Results of worse risk analyses^a^.

Result	ICBT^b^ group (worse=6%) and control group (worse=24%) (%)
RR^c^	26
RRR^d^	73
ARR^e^	17
NNT^f^	6

^a^Formula: RR=percentage of worse event ICBT group/control group; RRR=1–RR; ARR=percentage of worse event in control group–ICBT group; and NNT=1/ARR.

^b^ICBT: internet-based cognitive behavioral therapy.

^c^RR: relative risk.

^d^RRR: relative risk reduction.

^e^ARR: absolute risk reduction.

^f^NNT: number needed to treat.

## Discussion

### Principal Findings

This study evaluated the effectiveness of unguided ICBT for adolescents and young adults with subthreshold SAD through a multicenter RCT. The dropout rate in the intervention group was 9% (3/34), with an ICBT implementation rate of 91% (31/34) and a completion rate of 65% (22/34). The participants who conducted ICBT showed a significant reduction in social anxiety symptoms compared to those who did not receive treatment. Key indicators representing substantial symptom improvement, namely, treatment response rate (ICBT: 19/31, 61% vs control: 9/38, 24%; *P*=.003) and recovery rate (ICBT: 21/31, 68% vs control: 13/38, 34%; *P*=.008), demonstrated significant differences between the groups, with the intervention group showing better outcomes. No serious adverse events were observed in either group. Regarding deterioration events in social anxiety symptoms, there were 2 (6%) out of 31 cases in the intervention group and 9 (24%) out of 38 cases in the control group, but the difference was not statistically significant (*P*=.10). In summary, this study indicates that ICBT is an effective intervention approach for improving social anxiety symptoms in adolescents and young adults with subthreshold SAD.

### Comparison With Prior Work

A systematic review and meta-analysis of outcomes from 20 RCTs on SAD meeting clinical diagnostic criteria demonstrated that ICBT is effective in improving social anxiety symptoms, with a moderate effect size (Hedge *g*=0.55) [51]. In this study, unguided ICBT was shown to be sufficiently effective for subthreshold SAD in adolescents and young adults. This expands the evidence on CBT studies for SAD from several perspectives. The first perspective is the absence of therapist guidance. Many conventional ICBT studies for SAD have included therapist guidance [26,30]. Our results suggest that providing unguided ICBT to students with subthreshold SAD significantly reduces social anxiety symptoms measured by LSAS compared to the untreated group with a moderate effect size (Hedge *g*=0.66-0.66). The moderate effect size calculated in this RCT was comparable to a meta-analysis of 10 RCTs on face-to-face CBT for SAD [15]. Most of the participants assigned to the intervention group in this RCT achieved the treatment course of the Clark and Wells [14] model. Therefore, it appears that, even without guidance, significant improvements in social anxiety symptoms can be achieved with sufficient engagement.

Another perspective is related to the acceptance of ICBT for adolescents and young adults who do not meet the diagnostic criteria of SAD but are at high risk. As an application in clinical psychiatric care, a longitudinal cohort study has reported that ICBT enhances treatment adherence and reduces social anxiety symptoms in adults at risk for the onset of SAD [52]. Since SAD tends to manifest in adolescence [6], similar effects for subthreshold SAD may be observed in student support services in high schools and universities. Given the high likelihood of anxiety disorders persisting into adolescence [53], preventing the onset of mental disorders through early intervention in high-risk individuals is an important endeavor. Therefore, future research on subthreshold SAD in adolescents should consider conducting longitudinal cohort studies within the context of ICBT studies. Additionally, 23.3% of adolescents and young adults experience some form of anxiety, and among them, 39.1% use some form of health care service [54]. Only 108 (39.1%) of the 277 individuals sought medical help for their mental health issues, while the remaining 169 (60.9%) did not access healthcare. Since young individuals with social anxiety tend to avoid seeking help and contacting others [55], unguided ICBT may have the potential to meet their needs.

A previous preliminary study involving 17 adolescents with SAD who underwent ICBT reported high satisfaction with unguided ICBT through web-based questionnaires and semistructured interviews [56]. Participants who received guided ICBT in this preliminary investigation particularly appreciated the ability to access the ICBT program multiple times, allowing for repeated self-help. In this RCT, among the 34 participants in the intervention group, the majority (n=31, 91%) performed an ICBT module at least once. While 65% (n=31) completed the full ICBT course, those who completed the entire treatment course—up to module 9, corresponding to the Clark and Wells [14] model—accounted for 82% (n=28). According to these results, unguided ICBT is probably more acceptable for adolescents and young adults with subthreshold SAD.

Preliminary findings from our results also indicate that receiving complete self-help ICBT reduced the worsening of SAD by 73%. In addition to its short-term efficacy, unguided self-help ICBT may potentially prevent deterioration—that is, inhibiting the onset of SAD—in youths with subthreshold SAD. Even in the absence of guidance from CBT therapists, implementing ICBT based on the Clark and Wells [14] model may be beneficial for young adults and adolescents with SAD symptoms.

### Limitations

This study has several limitations that may increase the risk of bias. First, the primary outcome, the self-reported LSAS, was not blinded. The participants assigned to the intervention group may have positive expectations regarding the intervention, which could potentially affect the outcome. In the future, RCTs with blinded assessors should be conducted. Second, the control condition in this study was a no-treatment condition. To control for biases introduced by receiving the intervention or no treatment, future RCTs should consider using sham applications as psychological placebos to ensure blinding. Third, the evidence obtained in this RCT pertains to short-term effectiveness and cannot speak to the medium- to long-term effects. Particularly, to assess the effects on preventing the deterioration of social anxiety symptoms and preventing the onset of SAD, future intervention studies for subthreshold SAD should include a randomized cohort study with an observation period of several years.

### Conclusions

This multicenter RCT, conducted in Japan, has demonstrated that unguided ICBT can reduce social anxiety symptoms in adolescents and young adults with subthreshold SAD. Unguided ICBT appears to be a user-friendly intervention approach for supporting subthreshold SAD. Complete self-help ICBT may be a practical treatment approach that prevents deterioration in adolescents and young adults at high risk of developing SAD. Future research should incorporate study designs measuring long-term outcomes and focus on conducting cohort studies aimed at assessing the risk of clinically diagnosed SAD.

## Appendix

Multimedia Appendix 1 CONSORT-eHEALTH (V 1.6.1).

## Abbreviations

ANCOVA: analysis of covariance
ARR: absolute risk reduction
CBT: cognitive behavioral therapy
CONSORT: Consolidated Standards of Reporting Trials
GAD-7: Generalized Anxiety Disorder-7
ICBT: internet-based cognitive behavioral therapy
LSAS: Leibovitz Social Anxiety Scale
NNT: number needed to treat
OR: odds ratio
PHQ-9: Patient Health Questionnaire-9
QALY: quality-adjusted life years
QoL: quality of life
RCT: randomized controlled trial
RRR: relative risk reduction
SAD: social anxiety disorder
SPIN: Social Phobia Inventory
